# Frontier and Hot Topics of Pulsed Fiber Lasers via CiteSpace Scientometric Analysis: Passively Mode-Locked Fiber Lasers with Real Saturable Absorbers Based on Two-Dimensional Materials

**DOI:** 10.3390/ma15196761

**Published:** 2022-09-29

**Authors:** Wen Zhou, Xiuyang Pang, Hanke Zhang, Qiang Yu, Fangqi Liu, Wenyue Wang, Yikun Zhao, Yan Lu, Zixin Yang

**Affiliations:** 1 College of Systems Engineering, National University of Defense Technology, Changsha 410073, China; 2 College of Advanced Interdisciplinary Studies, National University of Defense Technology, Changsha 410073, China; 3 College of Meteorology and Oceanography, National University of Defense Technology, Changsha 410073, China; 4 Hubei Province Key Laboratory of Systems Science in Metallurgical Process, The State Key Laboratory for Refractories and Metallurgy, College of Science, Wuhan University of Science and Technology, Wuhan 430081, China; 5 Jiangxi Key Laboratory of Photoelectronics and Telecommunication, College of Physics and Communication Electronics, Jiangxi Normal University, Nanchang 330022, China; 6 School of Transportation Engineering, Jiangsu Shipping College, Nantong 226010, China

**Keywords:** fiber lasers, ultra-short pulses, passively mode-locked, saturable absorbers, 2D materials

## Abstract

Pulsed fiber lasers, with high peak power and narrow pulse widths, have been proven to be an important tool for a variety of fields of application. In this work, frontier and hot topics in pulsed fiber lasers were analyzed with 11,064 articles. Benefitting from the scientometric analysis capabilities of CiteSpace, the analysis found that passively mode-locked fiber lasers with saturable absorbers (SAs) based on two-dimensional (2D) materials have become a hot research topic in the field of pulsed fiber lasers due to the advantages of self-starting operation, high stability, and good compatibility. The excellent nonlinear optical properties exhibited by 2D materials at nanometer-scale thicknesses have become a particularly popular research topic; the research has paved the way for exploring its wider applications. We summarize the performance of several typical 2D materials in ultrafast fiber lasers, such as graphene, topological insulators (TIs), transition metal dichalcogenides (TMDs), and black phosphorus (BP). Meanwhile, we review and analyze the direction of the development of 2D SAs for ultrafast fiber lasers.

## 1. Introduction

Fiber lasers have attracted wide concern due to their compactness, high efficiency, high stability, low cost, and free maintenance compared to all-solid-state lasers. Among them, pulsed fiber lasers, with high peak power and narrow pulse widths, have been proven to be an important tool for a variety of fields of application, such as communications, industrial, and medical fields [1,2,3,4]. Especially in special applications requiring high average power, ultrashort pulse fiber laser sources can even compete with conventional Ti:sapphire solid-state counterparts due to their superior heat dissipation capabilities [5]. As a method of generating ultrafast pulses, the active mode-locking technique is complex and costly, and the pulse width is limited by the modulation frequency of the modulator. The passive mode-locking technique is an important method for generating ultrashort pulse lasers with the advantages of simple structure, easy start-up, and being alignment-free. Since the loss modulation of passive mode-locking is much faster than active mode-locking and its pulse width compression is more intense, passive mode-locking is becoming one of the most effective solutions for generating femtosecond lasers. However, with a conventional manual literature survey method, the Frontier and hot topics of pulsed fiber laser analysis are still limited by reading speed and time; bag data technology can expand the literature survey.

To date, scientometrics is an interdisciplinary discipline used for analysis and description that has become a powerful tool for assessing research performance by identifying articles that have been influential in the development of research in a field. With the development of the field of recent optics, the research papers related to pulsed fiber lasers are increasing. It is necessary to summarize and evaluate the relevant papers and research results in the field of pulsed fiber lasers using scientometric analysis to classify the research directions, promising and popular research areas, and development trends in the field. CiteSpace analysis software is a Java language-based visual analysis software for scientific papers developed by Dr. Chaomei Chen. The analysis of co-occurrence and co-citation of authors, keywords, and references in related literature is used to explore the current state of research in a field [6,7].

In recent decades, many investigations have been developed for the ultrashort pulse generation of fiber lasers based on passive mold-locking technology. Among them, the real saturable absorber (SA)-based ultrafast fiber lasers have gained a lot of attention because of their self-starting operation, high stability, low cost, and good compatibility advantages compared to artificial SAs such as nonlinear polarization rotation (NPR), nonlinear optical loop mirrors (NOLM), and nonlinear amplifying loop mirror (NALM) [8,9,10,11,12,13,14]. Semiconductor saturable absorber mirror (SESAM) is a well-established passive mode-locking device that has been widely used in solid state and fiber lasers [15,16,17,18]. However, the narrow saturable absorption bandwidth, long recovery time, and low damage threshold of SESAM have limited its further application. Therefore, novel real SAs with higher performance need to be developed to support the development of ultrafast lasers. Two-dimensional (2D) materials with excellent physical and chemical properties have attracted significant attention since the successful preparation of graphene [19]. In particular, the quantum confinement effect generated by the low-dimensional structure gives 2D materials more exceptional properties, such as outstanding nonlinear optical response [20]. In 2009, graphene was first applied as an SA in a passively mode-locked fiber laser to generate ultrashort pulses [21]. Since then, a large number of 2D materials have been developed and studied and they have exhibited tremendous application potential as SAs in fiber lasers. Until now, the most widely investigated 2D materials in the field of pulsed fiber lasers are graphene [22,23,24,25], topological insulators (TIs) [26,27,28], transition metal dichalcogenides (TMDs) [29,30,31], and black phosphorus (BP) [32,33,34]. Due to the broadband optical response of 2D materials, pulsed fiber lasers based on 2D materials can operate at different wavelengths, and related research work is mainly focused on 1 μm and 1.5 μm. The well-established fiber integration methods have brought a lot of convenience in preparing fiber SAs with unique properties, which has greatly promoted the development of passively mode-locked ultrafast fiber lasers based on 2D SA devices. In addition, passively mode-locked fiber lasers based on SA devices with simple structure, easy operation, and low cost are also the ideal platform for investigating optical solitons. There have been various SAs used in passively mode-locked fiber lasers, especially artificial SAs such as single-wall carbon nanotubes (CNTs) [35,36], graphene, and other 2D materials. Over the last decade, a large number of optical soliton studies have been investigated based on passively mode-locked fiber lasers [37,38,39]. The solitons are mainly grouped into conventional solitons [40], self-similar solitons [41], dissipative solitons [42], and dispersion management solitons [43]. In a soliton fiber laser, the soliton pulse does not change during propagation due to the delicate balance between linear and nonlinear effects in the optical medium, so the soliton is not disturbed by external signals, and the waveguide structure of the fiber provides a well distributed spatial quality of the output pulse. With the continuous development and exploration of optical solitons, it also promotes the further application of passively mode-locked ultrafast fiber lasers based on 2D SAs in industrial, imaging, and communication fields.

In this paper, a scientometric analysis was conducted using CiteSpace to explore the current status and research trends in the pulsed fiber lasers field over the last 6 years (2017–2022). Based on the literature references extracted from the Web of Science (WoS) database, the major hot studies of pulsed fiber lasers are reviewed. Finally, the current challenges and developments regarding pulsed fiber lasers are discussed.

## 2. Data Sources and Analysis Methods

In many previous review papers, descriptions of research hotspots, trends, and developments are often based on the authors’ systematic compilation of some literature items, and it is doubtful whether they are correct, insightful, and interpretable. The difference between this paper and other existing review papers was that it used scientometric analysis methods to mine the massive amount of co-cited literature data to provide a scientific analysis basis for identifying research hotspots and frontiers, evolutionary features, and paradigm evolution in the field of pulsed fiber lasers, thus solving the problem of lack of scientific analysis and interpretability in previous review articles when sorting out research hotspots, trends, and patterns.

The data for scientometric analysis came from the WoS core collection database, and the search term included “pulsed fiber lasers”, “fiber lasers soliton”, “pulse generation AND fiber”, “ultrashort pulse AND fiber”, “ultrafast AND fiber”, “mode-locking AND fiber”, “Q-switching AND fiber”, “semiconductor saturable absorber mirrors AND fiber”, “saturated absorbers AND fiber”, “modulation depth AND fiber”, “saturation intensity AND fiber”, “chirped AND fiber”, “(CPA OR chirped pulse amplification) AND fiber laser”, “optical solitons AND fiber”, “picosecond AND fiber”, “femtosecond AND fiber”, “millijoule AND fiber”, “nanosecond AND fiber”. The time span was set to from 2017 to 2022. The total number of records of data found was 14,317, including 11,064 articles, 17 corrections, 13 editorial materials, 5 letters, 18 meeting abstracts, 6 news items, 2907 proceedings papers, and 287 reviews. Finally, 11,064 articles were selected for analysis.

CiteSpace is a Java-based platform for the visualization of scientific bibliometric analysis software [44]. In this work, CiteSpace (5.8. R3) was used to conduct a scientometric analysis. The selected time period was from January 2017 to December 2022, with a time slice of 1 year and the rest of the settings as default. Specifically, we retrieved a large amount of citation data from the WoS core repository using the keyword pulsed fiber lasers, constructed a co-citation network of cited references in the field of pulsed fiber lasers using CiteSpace, and obtained the results of the scientometric analysis in this field by means of cluster analysis and timeline view. This allows us to answer the following questions: What is the state of cooperation in this field? What are the research hotspots and evolutionary features of the field? How are the research frontiers, knowledge base, and research paradigms evolving in the field? On this basis, we can conduct a literature review of the hotspots in this field to provide guidance for subsequent research.

## 3. Results

**Figure 1:**
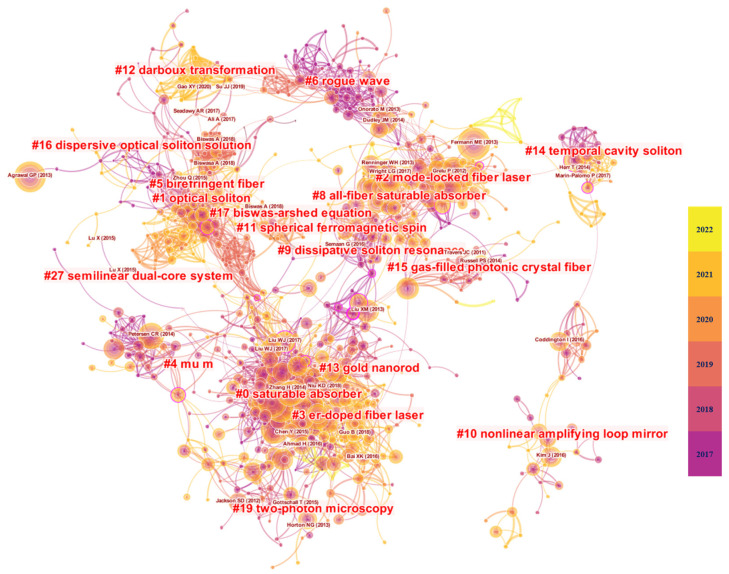
The co-citation network of reference from 2017 to 2022. In the literature co-citation network, the size of the nodes represents the size of the number of citations of the paper, and the color of the network nodes from dark to light indicates the evolution of the research from early to recent. The connection between two papers represents that the papers have a co-citation relationship, and the color of the connection represents the time when the two papers were first cited together. Based on the network clustering algorithm, the literature co-citation network was divided into 19 clusters. Based on the LLR algorithm, the tags of the clusters were extracted from the titles, keywords, and abstracts of the cited papers, which were represented as “# number tags”.

This paper performed a scientometric analysis using a literature co-citation network, which was constructed based on the co-citation relationships between papers, and its data source was the references cited by 11,064 cited papers during 2017–2022. The co-citation network of cited references is shown in Figure 1, and this network has 1089 nodes, 5383 connected edges, and a network density of 0.0091, with the largest one containing 865 nodes in the connected subgraph of the network.

Using the cluster analysis tool of CiteSpace, the cluster analysis results of the literature co-citation network were obtained, which included a total of 19 associations. Based on this, the paper extracted the subjects that can characterize the contents of the corresponding co-cited clusters from the titles, keywords, and abstracts of the cited literature of each cluster by using the Log-Likelihood Ratio (LLR) algorithm in CiteSpace. The clustering analysis reveals that the highly cited papers in the network are mainly concentrated in the three clusters of saturable absorber, optical solition, and mode-locked fiber laser, which represent three important research hotspots in the field of pulsed fiber lasers. They represent three important research hotspots in the field of pulsed fiber lasers, and a review of their current research status can help clarify the research development process in this field and provide guidance for subsequent research.

**Figure 2:**
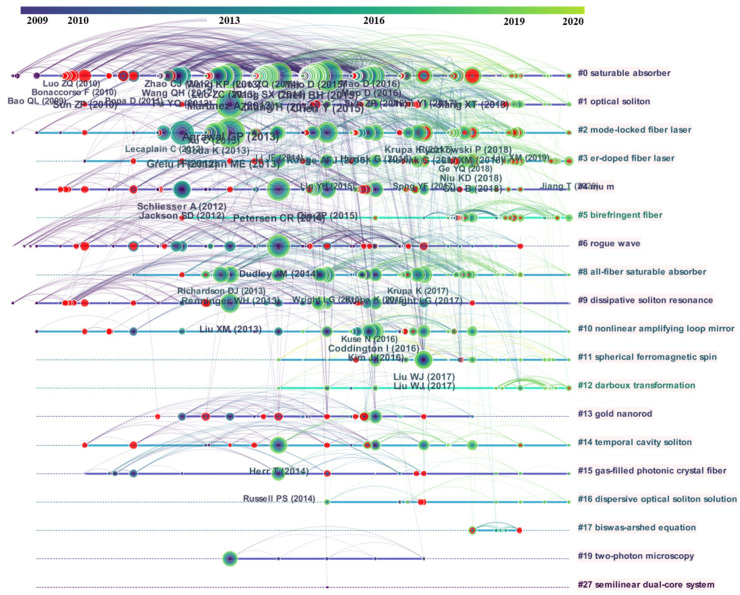
Timeline view of the co-citation network. In the timeline view, each node represents a paper, the size of the nodes represents the size of the number of citations of the paper, the color of the network nodes and the horizontal line from dark to light represent the evolution of the paper’s citation time from early to recent. It should be noted that the red node indicates that it is a burst literature, which plays an important role in this research field. The time of the literature is placed at the top of the view, and the further to the right, the more recent the literature is. In the timeline view, the same clusters of literature are placed on the same horizontal line. The timeline view provides a clear picture of the number of documents in each cluster and the temporal width of the study. More literature in a cluster means the resulting cluster area is more important, and a larger time span reflects a cluster having lasted longer. In addition, the comparison of the time span of the various types of literature on the timeline also allows for an analysis of the rise, boom, and decline of research in the field over time, and thus a discussion of the timing of scientific research.

Figure 2 shows the timeline view of the co-citation network. In the timeline view, the literature in the same clusters is placed on the same horizontal line. The timeline view provides a clear picture of the volume of literature in each cluster and the temporal width of the study. In addition, the comparison of the time span of the various types of literature on the timeline also allows for a discussion of the rise, boom, and decline process of research in the field over time. In Figure 2, we can see the volume of literature in each cluster and the time width of the research. For the three main clusters of saturable absorber, optical solition, and mode-locked fiber laser, we can see that the field of saturable absorber has gathered a large amount of research and the research duration is long, and its research boom period is between 2013 and 2016. The research duration of the optical solition field is weaker than that of the saturable absorber field, and a large number of studies are concentrated in 2013, but there are small-scale breakthroughs in 2015, 2017, and 2018. The mode-locked fiber laser field has a longer research duration than that of the saturable absorber field. The research duration in the field of mode-locked fiber laser is slightly weaker than that in the field of optical solition, but the aggregated research scale is more evenly distributed than that in the field of optical solition, and there are large-scale research breakthroughs from 2017 to 2019, and this trend continues until 2022, so it is worth tracking the research direction.

By analyzing the three largest clusters in the co-citation network, as shown in Table 1, it can be seen that the largest cluster (#0) has 199 members and a silhouette value of 0.878. It is labeled as a “saturable absorber” by both Log-Likelihood Ratio (LLR) and Latent Semantic Index (LSI), and as a “graded-index waveguide (2.62)” by MI. The most relevant citer to the cluster is “Guo B, 2018” [45]. The study highlights the important applications of different 2D materials in ultrafast lasers. In addition, the second largest cluster (#1) has 97 members and a silhouette value of 0.932. It is labeled as an “optical soliton” by both LLR and LSI, and as a “graded-index waveguide (1.34)” by MI. The most relevant citer to the cluster is “Fan, X.Y, 2019” [46]. The third largest cluster (#2) has 68 members and a silhouette value of 0.952. It is labeled as a “mode-locked fiber laser” by both LLR and LSI, and as “graded-index waveguide (1.68)” by MI. The most relevant citer to the cluster is “Krupa, K 2019” [47]. Passively mode-locked fiber lasers based on SAs are the ideal platform for investigating optical solitons. The unique nonlinear optical properties of 2D materials enable them to be used as outstanding SAs, and can be easily integrated into lasers. This advantage has further attracted extensive attention in the field of pulsed fiber lasers, making SAs based on 2D materials a hot research topic. Until now, various passively mode-locked fiber lasers have been developed by using the 2D SAs. We will briefly review these 2D materials in the next section of the article. Since we are dealing with scientometric data, the order of the SAs presented in the article is based on the first appearance in the literature of a specific category of 2D material as SA.

## 4. Passively Mode-Locked Fiber Lasers Based on 2D Materials

### 4.1. Passively Mode-Locked Fiber Lasers Based on Graphene

Graphene is a typical representative of two-dimensional materials and is one of the most widely studied materials. As an isomer of carbon atoms, graphene is a single layer of carbon atoms arranged in a two-dimensional honeycomb lattice [48,49,50]. Due to the pervasive optical absorption and zero bandgap, in principle, graphene is easily saturated with strong excitation in the visible to near-infrared region. Furthermore, it has the advantages of uniform nonlinear optical response and easy integration into the laser cavity. These properties give graphene huge potential for application as SA in fiber lasers.

**Figure 3:**
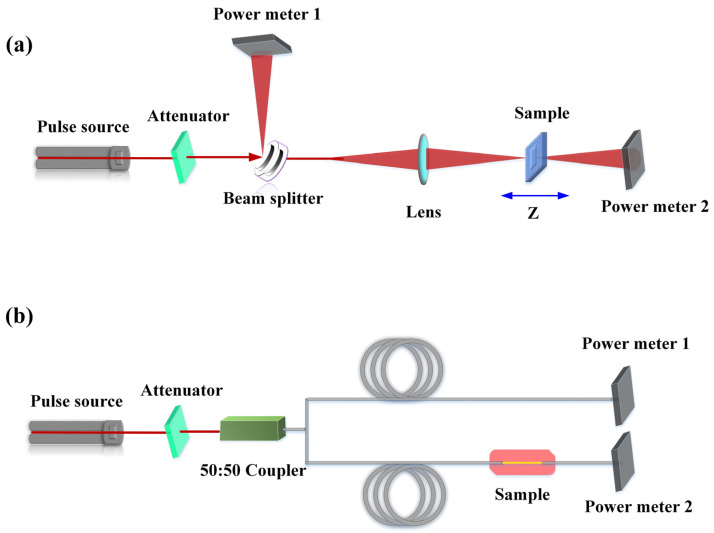
(**a**) Schematic of the Z-scan measurement setup. (**b**) Schematic of the two-arm measurement setup.

SA has three important parameters, i.e., modulation depth ($\alpha_{s}$), saturation intensity ($I_{s}$), and unsaturation loss ($\alpha_{ns}$). The relationship between the three parameters is expressed by the following equation:

(1) $$T=1-\frac{\alpha_{s}}{1+I/I_{s}}-\alpha_{ns}$$

where *I* and *T* are input optical intensity and transmittance. Currently, the typical methods for testing the saturable absorption properties of materials are Z-scan and dual-arm measurement methods [51,52]. The experimental setup for the Z-scan measurement is shown in Figure 3a, where an ultrafast laser source (pulse width in the order of ps or fs) is divided into two paths by a beam splitter: the measurement path and the reference path. In the reference light path, the light intensity is measured utilizing a power meter 1. In the measurement path, the pulsed light is focused by a lens onto the sample to be measured, which is mounted on a translation table that moves back and forth in the direction of the light (z-axis). As the sample moves, the spot size of the pulsed light on it varies, resulting in a change in energy density, which is measured by a power meter 2 after the light has passed through the sample. If there is no aperture in front of the power meter, this method is called open-aperture Z-scan measurement; otherwise, it is a closed-aperture Z-scan measurement. In addition to the Z-scan technique, two-arm measurement is also used as a simplified version of the measurement device to measure the saturated absorption properties of materials, and the experimental setup of the two-arm measurement method is shown in Figure 3b. In the measurement, all light is confined within the fiber, and after passing through an attenuator, the pulsed light is split into two paths: the reference path and the measurement path. After the pulsed laser passes through the sample to be measured, a power meter in the optical path is used for the measurement. By adjusting the input power of the pulsed light source, the intensity of the light interaction with the material can be changed. The nonlinear absorption characteristics of the sample can be obtained by comparing it with the power of the reference optical path.

**Figure 4:**
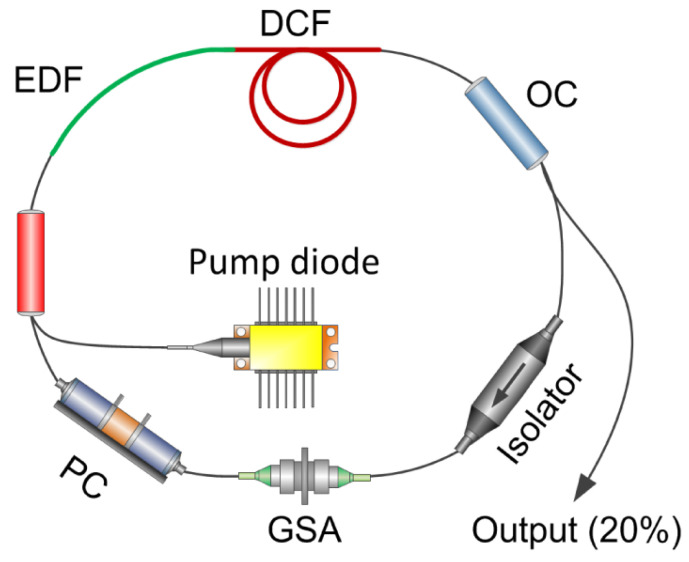
The setup of the stretched-pulse Er-doped mode-locked fiber laser (EDF—erbium-doped fiber; DCF—dispersion compensation fiber; OC—output coupler; GSA—graphene saturable absorber; PC—polarization controller). Reproduced from ref. [58]. Optical Society of America, 2015.

**Figure 5:**
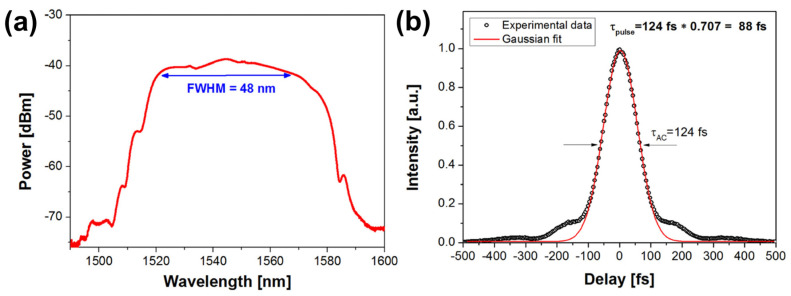
(**a**) The optical spectrum with FWHM of 48 nm. (**b**) The autocorrelation trace. Both measured at 110 mW pump power level. Reproduced from ref. [58]. Optical Society of America, 2015.

In 2009, researchers discovered that graphene has saturable absorption properties and can be used as an optical modulation device [21,22,23]. The mode-locked fiber lasers based on graphene SAs in the 1.5 μm sort were successfully realized in their works. The realized ultrafast lasers have pulse widths of 1.17 ps [21], 700 fs [22], and 415 fs [23], corresponding to repetition frequencies of 1.79 MHz, 6.95 MHz, and 6.84 MHz, respectively. Their research indicates that graphene has excellent ultrafast carrier dynamics and is an excellent SA with a fast response time for optical modulation. Compared to conventional SESAM and CNTs, graphene does not require bandgap optimization and diameter or chiral tuning, resulting in extremely efficient preparation. Due to its broadband saturable absorption, graphene can be used to implement broadband wavelength-tunable pulsed fiber lasers. In the last decade or so, a large number of passively mode-locked lasers based on graphene as a SA have been successfully realized and reported [53,54,55,56,57,58,59,60,61,62,63,64,65,66,67,68,69,70,71,72], as shown in Table 2. For graphene, passively mode-locked fiber lasers have been studied mainly for 1.5 μm and fewer studies have been conducted for 1, 2, and 3 μm. In 2015, Sotor et al. realized a stretched-pulse, mode-locked erbium (Er)-doped fiber laser at 1.5 μm based on graphene SA, and the all-fiber dispersion managed laser resonator with the repetition frequency of 21.15 MHz allows for Gaussian pulse generation with a pulse width of 88 fs. The experimental setup and the corresponding pulse data are shown in Figure 4 and Figure 5 [58]. In the same year, Purdie et al. combined a graphene mode-locked oscillator with an external compressor and achieved 29 fs pulses with 52 mW average power [64]. In 2022, based on graphene SA, mode-locked femtosecond pulses with different lengths of single-mode fiber (SMF) in an Er-doped fiber laser cavity were obtained by Abas et al. and the pulse width behavior varies from 820 fs to 710 fs against different cavity lengths [68].

**Figure 6:**
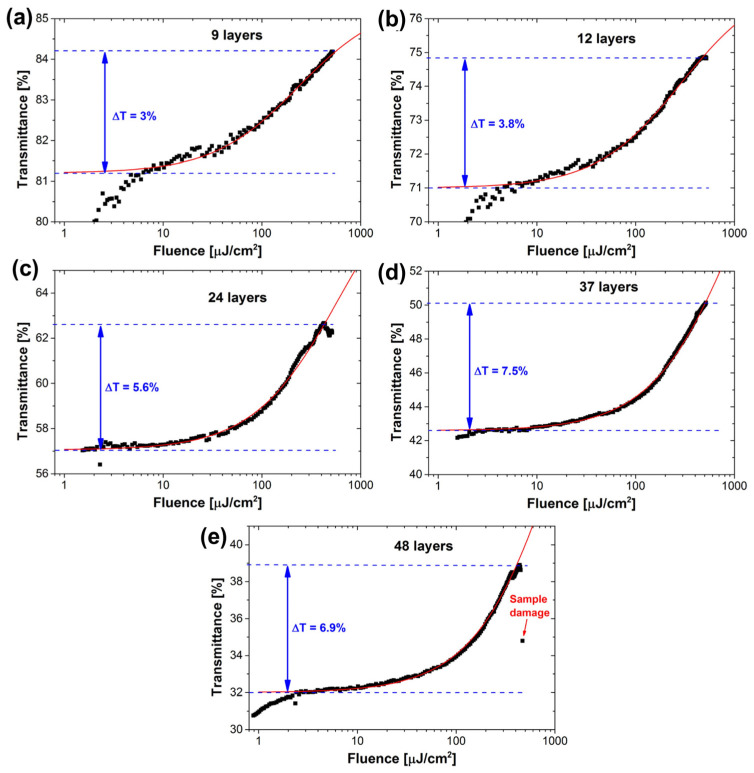
Measured power-dependent transmission of the fabricated multilayer graphene SAs: (**a**) 9 layers, (**b**) 12 layers, (**c**) 24 layers, (**d**) 37 layers, and (**e**) 48 layers. Reproduced from ref. [61]. Optical Society of America, 2015.

**Figure 7:**
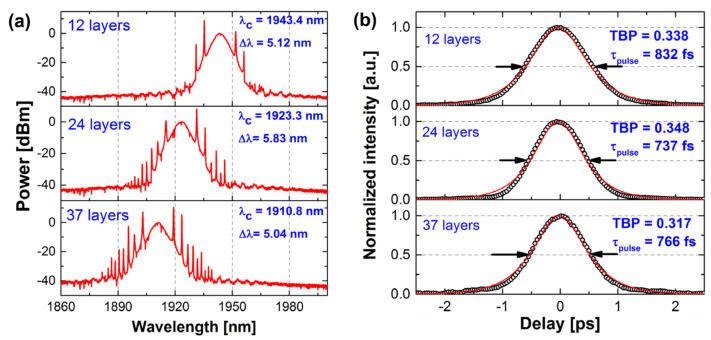
Measured optical spectra (**a**) and pulse durations (**b**) of the TDFL for different numbers of graphene layers in the SA. Reproduced from ref. [61]. Optical Society of America, 2015.

Moreover, the highest repetition frequency of the harmonic mode-locked laser implemented using 2D graphene SA is 2.22 GHz at 1.5 μm [65]. In the 1 μm range, mode locking of a ytterbium (Yb)-doped fiber laser with atomic multilayer graphene was experimentally demonstrated for the first time by Zhao et al. [54]. In their work, dissipative solitons with a duration of 580 ps at 1069:8 nm were generated. In the mid-infrared band, Sobon et al. fabricated a set of SAs with different numbers of graphene layers: 9, 12, 24, 37, and 48. In their work, each graphene SA with a different number was characterized in terms of nonlinear optical parameters (modulation depth, saturation intensity, saturation fluence), and the results are shown in Figure 6. Furthermore, a mode-locked laser at 2 μm was successfully realized using graphene SA with a different number in their work. Among them, the best performance (737 fs pulses with 5.82 nm bandwidth) was achieved with 24 layers, and the characterization results of the mold-locked pulses are shown in Figure 7 [61]. Yang et al. reported a broadband wavelength-tunable graphene mode-locked fiber laser at 2 μm [71]. In their work, the graphene film is tightly attached to the upper surface of the microfiber. This structure can be used not only as SA but also as a weakly polarized component. The operating wavelength of the mode-locking pulse can be tuned from 1880 nm to 1940 nm by adjusting the polarization controller (PC), and the minimum pulse width was 1.9 ps. Zhu et al. reported a mid-infrared Er-doped ZrF_4_-BaF_2_-LaF_3_-AlF_3_-NaF mode-locked fiber laser by using the multilayer graphene SA [72]. Mode-locked pulses at 2.8 µm with a pulse width of 42 ps at a repetition rate of 25.4 MHz, corresponding to a pulse energy of 0.7 nJ, were obtained. This series of studies demonstrated well the excellent broadband nonlinear modulation properties of graphene, and also promoted the development of passively mode-locked fiber lasers. It is important to note that graphene also has drawbacks, most significantly, its low on–off ratio and optical absorption, which can further limit its application in the field of optoelectronics [73]. Moreover, Malouf et al. found that the effective modulation depth of multilayer graphene is limited by two-photon absorption, which will affect its performance as an SA at longer wavelengths [74]. Therefore, it is necessary to continue to improve graphene properties or explore the novel nonlinear optical materials.

**Table 2:** Summary of passively mode-locked fiber lasers with graphene materials.

Materials	Gain Medium	Wavelength	Pulse Width	Repetition Rate	Pulse Energy	Ref.
	Yb-doped	1035 nm	6.5 ns	16.29 MHz	0.81 nJ	[53]
	Yb-doped	1069.8 nm	580 ps	0.9 MHz	0.41 nJ	[54]
	Er-doped	1567 nm	1.17 ps	1.79 MHz	—	[21]
	Er-doped	1590 nm	700 fs	6.95 MHz	3 nJ	[22]
	Er-doped	1576 nm	415 fs	6.84 MHz	7.3 nJ	[23]
	Er-doped	1565.3 nm	148 fs	101 MHz	15 pJ	[55]
	Er-doped	1559.12 nm	432.47 fs	25.51 MHz	0.09 nJ	[56]
	Er-doped	1565 nm	190 fs	42.8 MHz	0.09 nJ	[57]
	Er-doped	1545 nm	88 fs	21.15 MHz	71 pJ	[58]
	Er-doped	1567 nm	220 fs	15.7 MHz	83 pJ	[59]
Graphene	Er-doped	1561.6 nm	1.3 ps	6.99 MHz	7.25 nJ	[60]
	Er-doped	1559.34 nm	345 fs	54.28 MHz	38.7 pJ	[61]
	Er-doped	1560 nm	200 fs	22.9 MHz	—	[62]
	Er-doped	1553 nm	10 ps	8 MHz	0.125 nJ	[63]
	Er-doped	1550 nm	29 fs	18.67 MHz	2.8 nJ	[64]
	Er-doped	1560 nm	900 fs	2.22 GHz	—	[65]
	Er-doped	1560.7 nm	390 fs	2.44 MHz	—	[66]
	Er-doped	1563.5 nm	700 fs	12.905 MHz	24 nJ	[67]
	Er-doped	1556.2 nm	730 fs	11.19 MHz	—	[68]
	Er-doped	1556.9 nm	803.5 fs	18.7 MHz	14.4 pJ	[69]
	Tm-doped	1945 nm	205 fs	58.87 MHz	220 pJ	[70]
	Tm-doped	1900 nm	1.9 ps	19.7 MHz	0.1 nJ	[71]
	Tm-doped	2780 nm	42 ps	25.4 MHz	0.7 nJ	[72]

### 4.2. Passively Mode-Locked Fiber Lasers Based on TIs

TIs are a group of materials with topological electronic properties, such as Bi_2_Se_3_, Bi_2_Te_3_, and Sb_2_Te_3_ [75,76]. They behave internally as an insulator, but their surface contains gapless conductive states. Similar to graphene, TIs have strong spin–orbit interactions and have a symmetric Dirac cone energy band structure. In addition, TIs have unique electronic and optical properties due to the combination of strong spin–orbit coupling and time-reversal symmetry [77]. Their small indirect band gap of 0.2~0.3 eV gives them a broadband nonlinear response from visible to mid-infrared wavelengths. It was found that a member of the TIs, Bi_2_Te_3_, has a modulation depth of more than 70% at 1570 nm, higher than most 2D materials [51]. Furthermore, the ultra-fast relaxation time and low scattering loss also give it a huge advantage as a high-quality SA.

**Figure 8:**
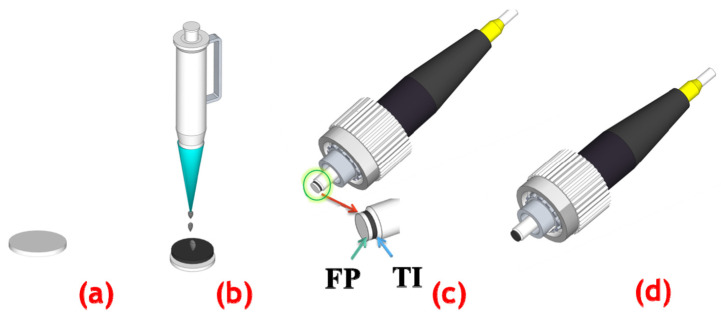
Procedures of the preparation of pure Bi_2_Se_3_-SA film. (**a**) Filter paper (FP) (GVWP02500 Millipore) with a pore size of 0.22 μm was immersed in deionized water until it soaked completely. (**b**) The Bi_2_Se_3_ water solution was dropwise added to the filter paper slowly. (**c**) Shear a small piece from the prepared Bi_2_Se_3_ filter paper and put it on the face of a fiber end-facet. (**d**) Put the fiber end-facet with the Bi_2_Se_3_ filter paper into acetone solution to remove the filter paper. Reproduced from ref. [81]. Optical Society of America, 2014.

**Figure 9:**
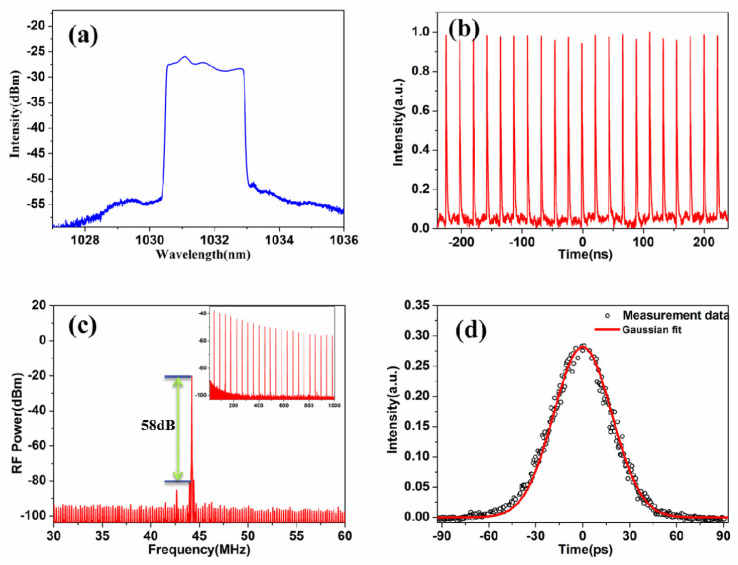
(**a**) Spectrum of the fiber laser at a pump power of 230 mW. (**b**) A typical mode-locking pulse train. (**c**) RF spectrum of the mode-locked laser. Inset: spectrum in 1 GHz span. (**d**) Autocorrelation trace. Reproduced from ref. [81]. Optical Society of America, 2014.

As TIs have attracted much attention due to their excellent optical properties, their potential as SA was further demonstrated by Zhao et al. in 2012 by conducting laser experiments [78,79]. In their works, a 1558 nm passively mode-locked ultrafast fiber laser with a pulse width of 1.21 ps was successfully achieved by using Bi_2_Te_3_ as SA [78]. In addition, a passively mode-locked Er-doped fiber laser with the Bi_2_Se_3_ as an SA was used. Stable soliton pulses with 1.57 ps pulse width at 1564.6 nm were obtained [79]. Their experimental results demonstrate that Bi_2_Te_3_ and Bi_2_Se_3_ nanosheets have ideal laser-locked optical properties and can be considered as another Dirac material type for SA, paving the way for TI-based ultrafast photonics. In 2014, Chi et al. experimentally demonstrated a 1.06 µm dissipative-soliton fiber laser based on a bulk-structured Bi_2_Te_3_ SA [80]. In their work, stable dissipative-soliton pulses with a composite temporal shape were obtained, and the temporal width of the output pulses was measured to be ~230 ps. Also in 2014, Dou et al. successfully achieved an all-normal-dispersion Yb-doped mode-locked fiber laser using a special method to paste a well-proportioned pure Bi_2_Se_3_ SA on a fiber end-facet [81]. Figure 8 shows the procedures of the preparation of the pure Bi_2_Se_3_-SA film. The corresponding results of the mode-locked fiber laser are shown in Figure 9. Mode-locked pulses with pulse energy of 0.756 nJ, a pulse width of 46 ps, and a repetition rate of 44.6 MHz were obtained. 

As an excellent broadband modulation device, research on the application of TIs in 2 μm band ultrafast fiber lasers has also been reported. A femtosecond mode-locked, all-fiberized laser that operates in the 2 μm region based on a Bi_2_Te_3_ SA was realized by Jung et al. in 2014 [82]. Ultrafast pulses with a temporal width of ~795 fs could readily be generated at a wavelength of 1935 nm. Subsequently, Lee et al. also successfully achieved a femtosecond mode-locked thulium–holmium (Tm-Ho) co-doped fiber-based Bi_2_Se_3_ SA with a pulse width of 853 fs [83]. Besides Bi_2_Te_3_ and Bi_2_Se_3_, Sb_2_Te_3_, as another typical representative of TIs, has also attracted extensive attention. For the first time, Sotor et al. used Sb_2_Te_3_ as an SA for efficient mode-locking of an Er-doped fiber laser at 1558.6 nm [84]. The pulse energy was at the level of 105 pJ with 1.8 ps pulse width and 4.75 MHz repetition rate. Subsequent ultrafast photonic applications of Sb_2_Te_3_ in the 1 μm and 2 μm bands have also been reported. In 2016, Kowalczyk et al. presented a study on a Sb_2_Te_3_-deposited side-polished fiber device as an SA for mode-locked fiber lasers in the 1 µm spectral range. The laser with 2 mm long Sb_2_Te_3_ absorber emitted 5.9 ps pulses with 4 mW of average output power [85]. In 2022, Lee et al. used Sb_2_Te_3_ as an SA to obtain stable mode-locked pulses in a Tm-Ho-doped fiber laser [86]. The ultrafast laser in their work had a repetition rate of 10.88 MHz and pulse width of 1.32 ps with a signal-to-noise ratio (SNR) value of about 61.21 dB. In the 3 μm band, a mid-infrared mode-locked fluoride fiber laser with TI Bi_2_Te_3_ nanosheets as the SA was presented by Yin and co-workers. In their work, The observed mold-locked pulse has a pulse repetition rate of 10.4 MHz, a pulse width of ~6 ps, and a center wavelength of 2830 nm. This work further demonstrates the promising application of 2D TIs in ultrashort laser generation. [87] Table 3 summarizes the performance of TIs in passively mode-locked fiber lasers [26,27,78,79,80,81,82,83,84,85,86,87,88,89,90,91,92,93,94,95,96,97,98,99,100,101,102,103]. The series of research works mentioned above have further demonstrated the excellent broadband optical response of TIs. However, compared to graphene, TIs are the slow saturable absorbing materials with a slower relaxation time. This limits their application in ultrafast photonics to some extent. In addition, as binary compounds, the preparation of TIs is more complicated, and in the meantime, their damage threshold is low. Therefore, there is a need to investigate enhancement of the damage threshold of TI SAs.

### 4.3. Passively Mode-Locked Fiber Lasers Based on TMDs

TMDs are a large material system including more than 40 different materials with the general formula MX_2_, where M stands for transition metals (e.g., Mo, W, Ti, Nb) and X stands for a group VI elements (e.g., S, Se, or Te) [104,105,106]. While graphene as well as TIs are being extensively studied, TMDs have also attracted significant interest because of their outstanding properties, such as switchable bandgap, higher third-order nonlinear optical response, ultrafast carrier dynamics, and so on. Compared with the zero band gap of graphene, the forbidden band structure of TMDs varies greatly with the thickness. Taking PtSe_2_ as an example, which has a layered CdI2-type structure, the monolayer PtSe_2,_ and the double layer PtSe_2_ have been proved to be semiconducting with a band gap of about 1.2 eV and 0.21 eV, respectively. As the number of layers increases, the bulk-structured PtSe_2_ becomes a semi-metal with zero band gap [107,108]. This special property associated with the layers gives TMDs the ability of broadband optical modulation with application performance comparable to or even better than zero-gap graphene. Meanwhile, the short recovery time (only a few picoseconds) of TMDs has great advantages in ultrafast pulsed laser generation. Therefore, a lot of research works of ultrafast fiber lasers have been carried out based on SA devices prepared from TMD materials.

**Figure 10:**
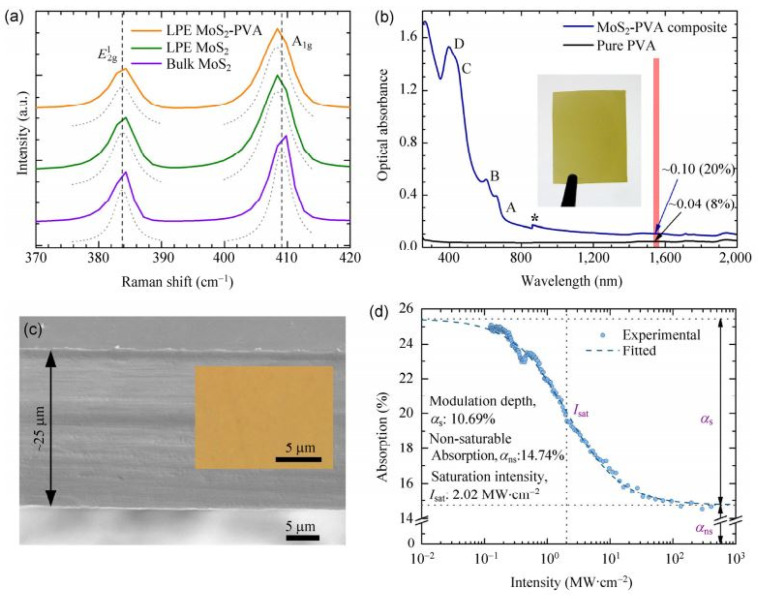
(**a**) Characterization of the MoS_2_-polymer composite film. (**a**) Raman spectra of the bulk MoS_2_ crystal, LPE MoS_2_ on Si/SiO_2_, and the MoS_2_-polymer composite film. (**b**) Linear optical absorption of the MoS_2_-PVA SA composite and pure PVA film of the same thickness. (**c**) SEM and optical micrograph (inset) image showing no aggregation/defect in the composite. The film thickness is ~25 µm. (**d**) Nonlinear optical absorption of the MoS_2_-PVA composite, measured via an open-aperture Z-scan at 1565 nm (~0.8 eV). Reproduced from ref. [111]. Springer Nature, 2015.

**Figure 11:**
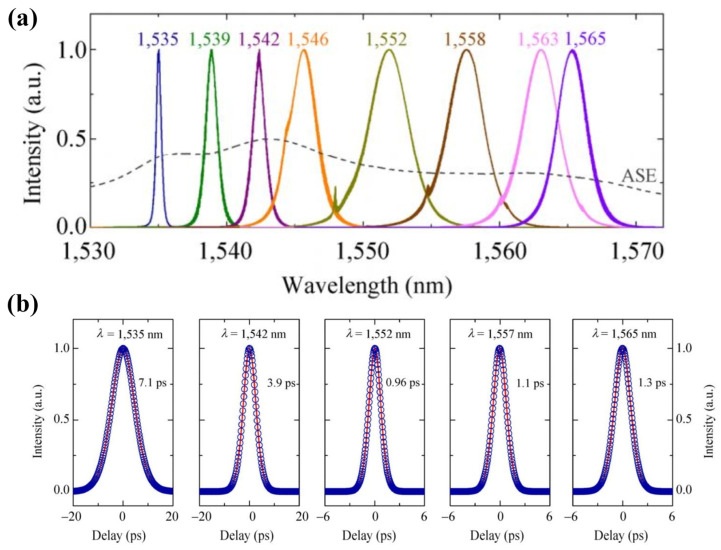
(**a**) Output spectra at eight representative wavelengths, continuous tuning from 1535 nm to 1565 nm. (**b**) Autocorrelation traces of the laser output at five representative wavelengths within the tunable operating range. Reproduced from ref. [111]. Springer Nature, 2015.

One of the first to be studied was MoS_2_. In 2013, researchers experimentally discovered that few-layer MoS_2_ has significant saturable absorption behavior, which is the key to achieving pulsed lasers [109]. Subsequently, Zhang et al. in 2014 characterized the nonlinear properties of MoS_2_ by employing an open-aperture Z-scan. An ultrafast fiber laser in the 1 μm band was successfully realized using MoS_2_ as SA. A stable mode-locked laser pulse, centered at 1054.3 nm, with a pulse duration of 800 ps was achieved [110]. In 2015, Zhang et al. demonstrated a wideband, tunable from 1535 nm to 1565 nm, ultrafast mode-locked fiber laser based on MoS_2_ SA with stable picosecond pulses [111]. The characterization of the MoS_2_-polymer composite film is shown in Figure 10. The results of the tuned spectra and ultrashort pulse measurements are shown in Figure 11. In the 2 μm band, Cao et al. reported an all-fiber passive mode-locking Tm-doped fiber laser that uses MoS_2_ as an SA material [112]. The central wavelength is 1926 nm and the pulse duration is 1.51 ps. Their works further illustrate the nonlinear optical properties of MoS_2_, especially at photon energies below the material band gap, suggesting new application opportunities for ultrafast photonics as well as other 2D TMDs (e.g., WS_2_, MoSe_2_, and WSe_2_). Like MoS_2_, layered WS_2_ also exhibits excellent nonlinear optical properties. In 2015, a 2D WS_2_-based SA for ultrafast photonic applications was demonstrated by Wu et al. [113]. By incorporating WS_2_-PVA SA into a fiber laser cavity, stable mode-locking operations were achieved at 1.5 μm. The short pulse duration was 595 fs, indicating the potential of WS_2_ in photonic applications. Then, Mao et al. reported the ultrafast optical applications of WS_2_ SA. The WS_2_ SAs are used to mode lock Er- and Yb-doped fiber lasers, producing trains of dissipative soliton [114]. The pulse duration was 21.1 ps and 630 ps at 1.55 µm and 1.06 µm, respectively. In addition, the band gap of WS_2_ nanosheets was reduced from 1.18 eV to 0.02 eV and 0.65 eV after the introduction of W-defects and S-defects, respectively, by theoretical calculations, which may help to improve the broadband saturable absorption characteristics of WS_2_.

Jung et al. demonstrated a mode-locked 1.94 μm fiber laser with a saturable absorption device based on a WS_2_-deposited side-polished fiber. Stable mode-locked pulses with a temporal width of ~1.3 ps were readily obtained at a wavelength of 1941 nm [115]. This work also further demonstrates the excellent broadband saturable absorption properties of WS_2_. In addition, mode-locked fiber lasers based on other layered TMDs (such as MoSe_2_, Wse_2_, SnS_2_, ReS_2_, etc.) have been reported successively. Table 4 summarizes the performance of several typical TMD materials for ultrafast fiber laser applications [29,30,31,110,111,112,113,114,115,116,117,118,119,120,121,122,123,124,125,126,127,128,129,130,131,132,133,134,135,136,137,138,139,140,141,142,143,144]. These studies show that TMDs are a promising layer material that can be used as SA for laser mode-locking. If precise tuning of their forbidden band width can be achieved, then this material will be used in a wider range of applications in optoelectronics.

### 4.4. Passively Mode-Locked Fiber Lasers Based on BP

**Figure 12:**
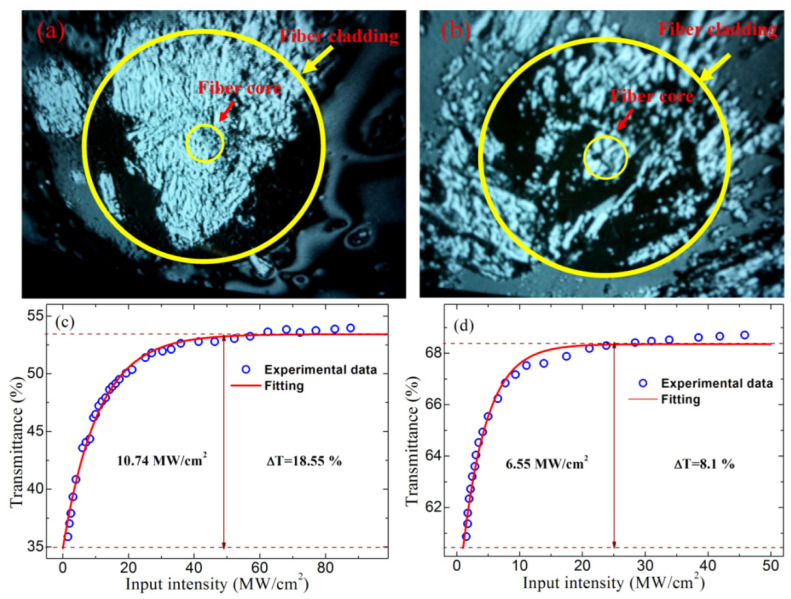
Optical image of the fiber end-facet (fiber cladding diameter of 125 μm, fiber core diameter of 9 μm) covered with relatively thick (**a**) and thin (**b**) BP and the measured saturable absorption data and its corresponding fitting curve of relatively thick (**c**) and thin (**d**) BP. Reproduced from ref. [32]. Optical Society of America, 2015.

**Figure 13:**
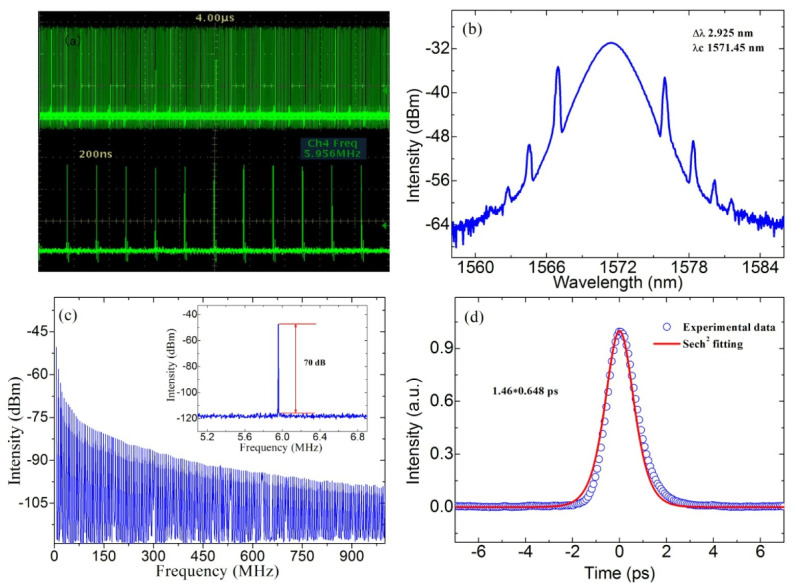
Typical Mode-locking output: (**a**) Output pulse train. (**b**) the corresponding spectrum. (**c**) the measured autocorrelation trace and its fitting curve. (**d**) RF spectrum. Inset: RF spectrum in 1 GHz span. Reproduced from ref. [32]. Optical Society of America, 2015.

BP is another material that has received significant attention, and is the most stable isomer of phosphorus at room temperature. Similar to graphene, BP is a stable six-atom chain ring structure formed by each phosphorus atom being linked to three adjacent phosphorus atoms. Moreover, the structure of BP is folded, which reduces its symmetry and introduces an angle-dependent nonlinearity. Like TMDs, it is a high mobility layered semiconductor with a band gap dependent on the number of layers, ranging from 0.3 eV (bulk) to 2.0 eV (monolayer) [145,146]. In addition, BP has direct transitions in all thicknesses and shows more absorption compared to TMDs, which should be more suitable for optoelectronic applications. In particular, the band gap of BP is located between graphene and the TMD semiconductor, so BP is considered a natural candidate to fill the “gap” between semimetallic graphene and wide band gap TMDs. It is worth mentioning that BP always maintains a direct band gap regardless of thickness variation, thus ensuring ultrafast electron relaxation performance. This unique band gap advantage of BP allows it to be better used for broadband optical applications, especially in the near-, and middle-infrared range. In 2015, the photovoltaic properties of BP were reported and attracted a lot of attention [147]. Lu et al. first experimentally demonstrated the ultrafast nonlinear optical response of multilayer BP nanosheets with the Z-scan measurement technique [148]. This work demonstrates that BP is a promising material for broadband nonlinear optics, especially in the long wavelength range. In 2015, Chen et al. successfully achieved a 1.5 μm mode-locked fiber laser using a novel SA based on mechanical stripping of BP [32]. Mode-locked pulses with a maximum pulse energy of 94.3 nJ and a pulse duration of 648 fs were obtained by integrating BP-based SA devices into an all-fiber Er-doped fiber laser cavity. Figure 12 shows the optical image of the fiber end-facet and the measured saturable absorption data of BP. The corresponding ultrafast pulse characterization is shown in Figure 13.

**Figure 14:**
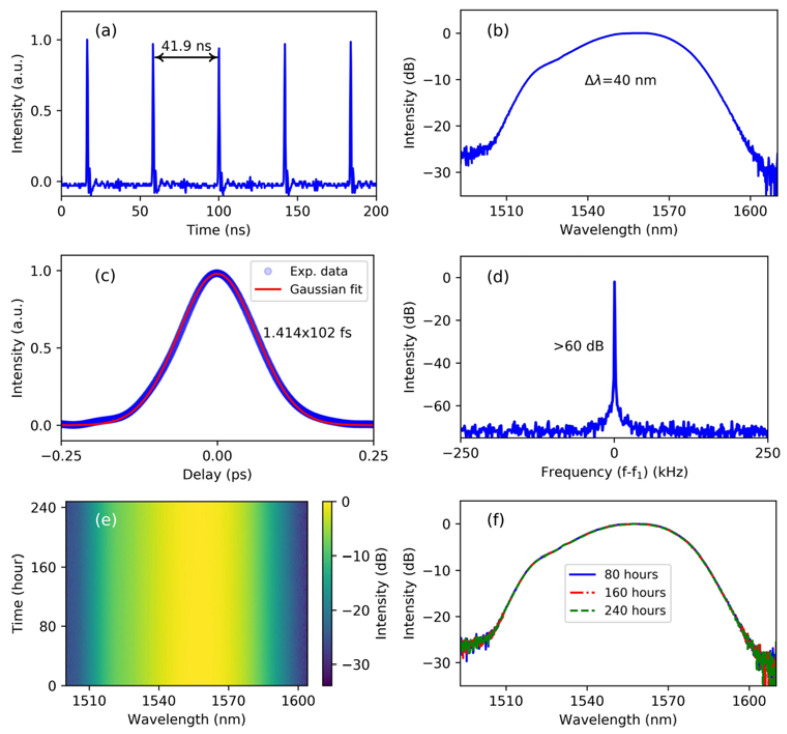
(**a**) Oscilloscope trace of the laser output. (**b**) Optical spectrum. (**c**) Autocorrelation trace with a Gaussian fit. (**d**) Radio frequency spectrum. (**e**) Spectra of long-term stable operation over 240 h. (**f**) Spectra acquired after 80 h (blue curve), 160 h (red dot line), and 240 h (green dot line), respectively. Reproduced from ref. [152]. Optical Society of America, 2018.

Also in 2015, Sotor et al. demonstrated that the saturation absorption of BP is polarization-sensitive, while a mode-locked fiber laser was successfully realized using mechanically peeled ~300 nm-thick BP transferred to the fiber core (which improved its transmittance by 4.6%) [149]. The generated optical solitons with the 10.2 nm bandwidth and 272 fs duration were centered at 1550 nm. At other bands, as a broadband SA, ultrafast lasers of BP are also widely studied. Sotor et al. also reported the usage of BP as an SA for the mode-locking of a Tm-doped fiber laser [150]. The mode-locked operation which is centered at 1910 nm has a pulse duration of 739 fs. This shows that BP exhibits saturable absorption in the 2 μm wavelength range and supports ultrashort pulse generation. In 2016, Hisyam et al. demonstrated the generation of mode-locked pulses from a double-clad Yb-doped fiber laser employing a BP SA [151]. A pulse duration of 7.54 ps, a repetition rate of 13.5 MHz, and maximum pulse energy of 5.93 nJ were realized. Since then, ultrafast laser applications of BP in the near- to mid-infrared have been widely reported, greatly contributing to the development of optical modulation devices based on 2D materials. Jin et al. used a scalable and highly controllable inkjet printing technique to fabricate BP SA with very excellent nonlinear characteristics. They also successfully realized a mode-locked fiber laser with a pulse width of 102 fs using BP SA, which is the shortest pulse achieved so far based on BP SA [152]. The experimental results are shown in Figure 14. In the mid-infrared band, Qin et al. reported a passively mode-locked Er:ZBLAN fiber laser with the as-prepared BP SA mirror at a wavelength of 2783 nm, which delivers a repetition rate of 24.27 MHz and a pulse duration of 42 ps [153]. Their work demonstrates the potential of BP as an excellent SA for applications in mid-infrared ultrafast photonics. The passively mode-locked fiber lasers based on BP SA are shown in Table 5 [32,149,150,151,152,153,154,155,156,157,158,159,160,161,162,163,164,165,166,167,168]. It is worth noting that although BP has achieved a wealth of research results, it is prone to oxidation in air, and further exploration of methods to stabilize it is necessary.

## 5. Conclusions and Outlook

The frontiers and hotspots of pulsed fiber lasers for passively mode-locked fiber lasers with real material SAs were analyzed using the method of scientometric analysis in CiteSpace. We found that the most important research hotspot in the field of pulsed fiber laser is “saturable absorber”. Moreover, based on a generalization of the literature, we found the importance of 2D materials in the development of SA. Undoubtedly, as a research hotspot in the field of pulsed fiber lasers, passively mode-locked fiber lasers based on 2D materials have gained wide reports. As the new type of SAs different from SESAM, the 2D material-based optical modulation devices also have unique advantages in enhancing laser performance. The excellent nonlinear optical properties exhibited by 2D materials at nanometer-scale thicknesses in particular have unique advantages in enhancing laser performance; this has paved the way for exploring wider applications. This paper provides an introduction to several typical 2D materials, and provides a summary of the laser performance achieved by SAs based on 2D materials, such as graphene, TIs, TMDs, and BP. In general, different 2D materials have their own advantages and disadvantages. While continuing to optimize the performance of typical materials, we also need to explore other new 2D materials. We believe that shortly, pulsed lasers based on various new nanomaterials will be more widely used for ultrafast photonic applications. Meanwhile, with the increasingly active research on 2D materials, passively mode-locked fiber lasers based on 2D SAs will be further developed. Future research efforts should focus on further improving the performance of ultrafast fiber lasers (e.g., the output power, repetition rate, pulse duration, and pulse energy), while also considering the long-term stability of material devices, providing better conditions for large-scale industrial applications. We look forward to seeing the emerging 2D SA-based ultrafast fiber lasers with excellent performance being widely used in various applications in society in the near future.

## Figures and Tables

**Table 1:** Summary of the largest 3 clusters.

Cluster ID	Size	Silhouette ^1^	Label (LSI) ^2^	Label (LLR) ^3^	Label (MI) ^4^	Average Year ^5^
0	199	0.878	saturable absorber	saturable absorber (18,479.61, 1.0 × 10^−4^)	graded-index waveguide (2.62)	2014
1	97	0.932	optical soliton	optical soliton (12,202.86, 1.0 × 10^−4^)	graded-index waveguide (1.34)	2016
2	68	0.952	mode-locked fiber laser	mode-locked fiber laser (5738.23, 1.0 × 10^−4^)	graded-index waveguide (1.68)	2015

^1^ Silhouette value is a parameter proposed by Kaufman and Rousseeuw in 1990 for evaluating the effect of clustering. The clustering is evaluated by measuring the homogeneity of the network. The closer the Silhouette value is to 1, the higher the homogeneity of the network reflected. The clustering result has high confidence when the Silhouette value is 0.7. The clustering result can be considered reasonable when the Silhouette value is above 0.5. ^2^ Log-Likelihood (LLR), Latent Semantic Index (LSI), and Mutual Information (MI) are three unsupervised learning algorithms for textual topic model mining. CiteSpace uses these three algorithms to extract clustering labels from the titles, keywords, and abstracts of the cited documents. Among them, LLR is a log-likelihood algorithm, LSI is a latent semantic indexing algorithm, and MI is a mutual information algorithm. Concerning the LLR algorithm, the basic use of LLR in text computing is described by Ted Dunning in “Accurate Methods for the Statistics of Surprise and Coincidence”. It is simple to implement, effective, and scalable on large scale data, and can be used well to calculate the similarity between text content and topics, so CiteSpace uses LLR to mine text topics. ^3^ Concerning the LSI algorithm, also called Latent Semantic Analysis (LSA), it is a very important class of technical ideas in the field of information retrieval. CiteSpace uses LSI to mine textual topics. ^4^ Mutual Information (MI) is how much information about X can be obtained by observing Y. ^5^ We conducted a scientometric analysis based on the co-citation network of cited references, which is constructed based on the co-citation relationship between papers, and its data source is the literature cited by the citing literature from 2017 to 2022. Since the publication time of the cited literature will be earlier than the citing literature, i.e., the reference cited in a particular article must have been published before this article, although we retrieved literature from 2017 to 2022, the average year of the literature in the clusters in the co-citation network may be before this time period. The “Average Year” in Table 1 represents the average of publication time in each cluster, where the three largest clusters are 2014, 2016, and 2015, indicating the average year of publication in these three clusters. We can consider them as the main time of the origin of the study.

**Table 3:** Summary of passively mode-locked fiber lasers with TI materials.

Materials	Gain Medium	Wavelength	Pulse Width	Repetition Rate	Pulse Energy	Ref.
	Yb-doped	1057.82 nm	230 ps	1.44 MHz	0.6 nJ	[80]
	Yb-doped	1064.47 nm	960 ps	1.11 MHz	——	[88]
	Er-doped	1547 nm	600 fs	15.11 MHz	53 pJ	[90]
	Er-doped	1560.8 nm	286 fs	18.55 MHz	0.03 nJ	[91]
	Er-doped	1562.4 nm	320 fs	17.34 Hz	32 pJ	[92]
Bi_2_Te_3_	Er-doped	1555.9 nm	630 fs	773.85 MHz	——	[93]
	Er-doped	1558.5 nm	2.49 ps	2.04 GHz	——	[94]
	Er-doped	1561.48 nm	0.78 ps	24 MHz	247.1 pJ	[95]
	Er-doped	1558 nm	1.21 ps	1.21 MHz	——	[78]
	Tm-Ho-doped	1935 nm	795 fs	27.9 MHz	——	[82]
	Ho-Pr-doped	2830 nm	6 ps	10.4 Mhz	8.6 nJ	[87]
	Yb-doped	1031.7 nm	46 ps	44.6 MHz	0.76 nJ	[81]
	Yb-doped	1038.5 nm	380 ps	16 MHz	1.06 nJ	[89]
	Er-doped	1564.6 nm	1.57 ps	1.21 MHz	——	[79]
Bi_2_Se_3_	Er-doped	1557.5 nm	660 fs	12.14 MHz	0.14 nJ	[96]
	Er-doped	1562.4 nm	630 fs	23.3 MHz	15.6 pJ	[97]
	Er-doped	1571 nm	579 fs	12.54 MHz	127 pJ	[98]
	Er-doped	1560.88 nm	1.754 ps	3.125 GHz	4.5 pJ	[99]
	Tm-Ho-doped	1912.12 nm	853 fs	18.37 MHz	——	[83]
	Yb-doped	1047.1 nm	5.9 ps	19.28 MHz	0.21 nJ	[85]
	Er-doped	1558.6 nm	1.8 ps	4.75 MHz	0.105 nJ	[84]
	Er-doped	1565 nm	128 fs	22.32 MHz	44.8 pJ	[100]
	Er-doped	1556 nm	449 fs	22.13 MHz	39.6 pJ	[26]
Sb_2_Te_3_	Er-doped	1568.6 nm	195 fs	33 MHz	0.27 nJ	[27]
	Er-doped	1561 nm	270 fs	38.54 MHz	29 pJ	[101]
	Er-doped	1558 nm	167 fs	25.38 MHz	0.21 nJ	[102]
	Tm-doped	1930.07 nm	1.24 ps	14.52 MHz	8.96 nJ	[103]
	Tm-Ho-doped	1902.06 nm	1.32 ps	10.87 MHz	283.34 pJ	[86]

**Table 4:** Summary of passively mode-locked fiber lasers with TMD materials.

Materials	Gain Medium	Wavelength	Pulse Width	Repetition Rate	Pulse Energy	Ref.
	Yb-doped	1054.3 nm	800 ps	6.58 MHz	——	[110]
	Yb-doped	1090 nm	21.84 ps	13.2 MHz	1.48 nJ	[116]
	Er-doped	1569.5 nm	710 fs	12.09 MHz	0.147 nJ	[29]
	Er-doped	1556.8 nm	3 ps	2.5 GHz	2 pJ	[30]
	Er-doped	1573.7 nm	630 fs	27.1 MHz	0.141 nJ	[31]
MoS_2_	Er-doped	1552 nm	960 fs	12.99 MHz	——	[111]
	Er-doped	1561 nm	246 fs	101.4 MHz	1.2 nJ	[117]
	Er-doped	1567.7 nm	1.4 ps	5.78 MHz	——	[118]
	Er-doped	1563 nm	2.17 ps	0.987 MHz	——	[119]
	Er-doped	1535 nm	1.07 ps	13 MHz	——	[120]
	Tm-doped	1926 nm	1.51 ps	13.9 MHz	——	[112]
	Tm-doped	1915.5 nm	1.25 ps	18.72 MHz	——	[121]
	Yb-doped	1063 nm	630 ps	5.57 MHz	13.6 nJ	[114]
	Yb-doped	1030.3 nm	2.5 ns	2.84 MHz	2.82 nJ	[122]
	Yb-doped	1029.1 nm	59 ps	19.03 MHz	——	[123]
	Er-doped	1572 nm	595 fs	——	——	[113]
	Er-doped	1565 nm	21.1 ps	8.05 MHz	2.2 nJ	[114]
	Er-doped	1560.1 nm	325 fs	30.91 MHz	——	[123]
WS_2_	Er-doped	1560 nm	288 fs	41.4 MHz	0.04 pJ	[124]
	Er-doped	1563.8 nm	808 fs	19.57 MHz	0.1336 nJ	[125]
	Er-doped	1565 nm	332 fs	31.11 MHz	14 pJ	[126]
	Er-doped	1560 nm	605 fs	8.83 MHz	1.14 nJ	[127]
	Er-doped	1540 nm	67 fs	135 MHz	——	[128]
	Er-doped	1560 nm	395 fs	19.57 MHz	76.6 pJ	[129]
	Tm-doped	1941 nm	1.3 ps	34.8 MHz	172 pJ	[115]
	Er-doped	1555.6 nm	737 fs	3.27 GHz	7 pJ	[130]
MoSe_2_	Er-doped	1560 nm	580 fs	8.8 MHz	91.3 pJ	[131]
	Er-doped	1552 nm	207 fs	64.56 MHz	——	[132]
	Tm-doped	1943.35 nm	980 fs	23.53 MHz	0.39 nJ	[133]
	Er-doped	1557.6 nm	1.25 ps	5.25 MHz	——	[134]
Wse_2_	Er-doped	1557.4 nm	163.5 fs	63.13 MHz	451 pJ	[135]
	Er-doped	1555.2 nm	698.5 fs	23.95 MHz	0.21 nJ	[136]
	Tm-doped	1863.96 nm	1.16 fs	11.36 MHz	2.9 nJ	[137]
	Yb-doped	1031 nm	282 ps	3.76 MHz	——	[138]
SnS_2_	Er-doped	1561 nm	1.63 ps	4.40 MHz	——	[138]
	Er-doped	1562.01 nm	623 fs	29.33 MHz	41 pJ	[139]
	Tm-doped	1910 nm	——	1.99 MHz	——	[138]
	Er-doped	1558.6 nm	1.6 ps	5.48 MHz	73 pJ	[140]
	Er-doped	1565 nm	2.549 ps	1.896 MHz	37 pJ	[141]
ReS_2_	Er-doped	1564 nm	1.25 ps	3.43 MHz	——	[142]
	Er-doped	1550 nm	220 fs	16.26 MHz	——	[143]
	Tm-doped	1970.65 nm	893 fs	26.1 MHz	——	[144]

**Table 5:** Summary of passively mode-locked fiber lasers with BP materials.

Materials	Gain Medium	Wavelength	Pulse Width	Repetition Rate	Pulse Energy	Ref.
	Yb-doped	1085.58 nm	7.54 ps	13.5 MHz	5.93 nJ	[151]
	Yb-doped	1030.6 nm	400 ps	46.3 MHz	0.70 nJ	[154]
	Er-doped	1571.45 nm	648 fs	5.96 MHz	2.3 nJ	[32]
	Er-doped	1560.5 nm	272 fs	28.2 MHz	——	[149]
	Er-doped	1555 nm	102 fs	23.9 MHz	0.08 nJ	[152]
	Er-doped	1556.5 nm	940 fs	9.46 MHz	——	[155]
	Er-doped	1558.14 nm	2.18 ps	15.59 MHz	——	[156]
	Er-doped	1569.24 nm	280 fs	60.5 MHz	——	[157]
	Er-doped	1559.5 nm	670 fs	8.77 MHz	——	[158]
BP	Er-doped	1560.7 nm	570 fs	6.88 MHz	0.74 nJ	[159]
	Er-doped	1558.8 nm	805 fs	3.82 MHz	——	[160]
	Er-doped	1558.7 nm	786 fs	14.7 MHz	0.11 nJ	[161]
	Er-doped	1562.8 nm	291 fs	10.36 MHz	——	[162]
	Er-doped	1555 nm	687 fs	37.8 MHz	——	[163]
	Er-doped	1562 nm	635 fs	12.5 MHz	——	[164]
	Tm-doped	1910 nm	739 fs	36.8 MHz	40.7 pJ	[150]
	Tm-Ho-doped	1898 nm	1.58 ps	19.2 MHz	440 pJ	[165]
	Ho-doped	2094 nm	1.3 ps	290 MHz	0.39 nJ	[166]
	Er-doped	2783 nm	42 ps	24.27 MHz	25.5 nJ	[153]
	Er-doped	2771.5 nm	——	27.4 MHz	0.23 nJ	[167]
	Er-doped	3489 nm	——	28.91 MHz	1.38 nJ	[168]

## Data Availability

Data are contained within the article.
